# Retraction: Synthesis and characterization of Co/Ti layered double hydroxide and its application as a photocatalyst for degradation of aqueous Congo Red

**DOI:** 10.1039/d0ra90027c

**Published:** 2020-04-06

**Authors:** Laura Fisher

**Affiliations:** Royal Society of Chemistry Thomas Graham House, Science Park, Milton Road Cambridge UK advances-rsc@rsc.org

## Abstract

Retraction of ‘Synthesis and characterization of Co/Ti layered double hydroxide and its application as a photocatalyst for degradation of aqueous Congo Red’ by Priyadarshi Roy Chowdhury and Krishna G. Bhattacharyya, *RSC Adv.*, 2015, **5**, 92189–92206.

The Royal Society of Chemistry hereby wholly retracts this *RSC Advances* article due to concerns with the reliability of the data in the published article.

The XPS data in Fig. 4B, E and F have been duplicated in another publication, but reported as a different material.^1^

A repeating segment can be observed in the TEM image presented in Fig. 10C, which indicates that this image has been manipulated.

There are unexpected similarities in the baseline of the EDX spectrum in Fig. 10F and the EDX spectra in other publications, which have all been reported as different materials.^1–3^

There are repeating motifs within the AFM image in Fig. 10G, which indicates that this image has been manipulated. Many of these motifs can also be observed in an AFM image in another publication, but representing a different material.^2^

The image in Fig. 10I is unreliable as it has subsequently been reused in unpublished material to represent different materials.

The FTIR data presented in Fig. 16B (blue, red and green spectra) illustrate duplication of data, given that these experiments were reported under different reaction conditions.

Given the number and significance of the concerns about the validity of the data, the findings presented in this paper are no longer reliable.

Priyadarshi Roy Chowdhury and Krishna G. Bhattacharyya were informed about the retraction of the article but did not respond.

Signed: Laura Fisher, Executive Editor, *RSC Advances*

Date: 12^th^ March 2020

## Supplementary Material
